# The Impact of Nutritional Condition and Compression Treatment on Venous Ulcer Recovery: A Systematic Review

**DOI:** 10.7759/cureus.57407

**Published:** 2024-04-01

**Authors:** Arwa T Alsharif, Omar I Alanazi, Rayan A Alqarni, Hawazen O Alahmadi, Lamis A Alassiri, Shahad A Alamri, Razan M Khalifa, Eman A Alshehab, Rahmah B Alzahrani, Abdullah Al Wahbi

**Affiliations:** 1 Department of Medicine, Batterjee Medical College, Jeddah, SAU; 2 Department of Medicine, King Saud Bin Abdulaziz University for Health Sciences, Riyadh, SAU; 3 Department of Medicine, Imam Mohammad Ibn Saud Islamic University (IMSIU), Riyadh, SAU; 4 Department of Medicine, Taibah University, Medina, SAU; 5 Department of Medicine, King Abdulaziz University, Jeddah, SAU; 6 Department of Medicine, Umm Al-Qura University, Mecca, SAU; 7 Department of Clinical Nutrition, King Fahd Hospital, Medina, SAU; 8 Department of Vascular Surgery, King Abdulaziz Medical City Riyadh, Riyadh, SAU; 9 Department of Surgery, King Saud University for Health Sciences, Riyadh, SAU

**Keywords:** compression bandages, compression therapy, malnutrition, nutrition assessment, undernutrition, varicose ulcer, venous leg ulcer, venous ulcer

## Abstract

Venous ulcers are open wounds commonly associated with chronic venous insufficiency. Each patient’s healing process is unique, and factors like nutrition and compression therapy can affect it. Compression therapy and optimal nutritional status can assist in improving venous blood circulation, decreasing swelling, and promoting wound healing. This in-depth review looks at all the recent research on how nutrition and compression therapy can help heal venous ulcers, aiming to develop evidence-based guidelines for improving treatment outcomes.

The systematic review, registered in the International Prospective Register of Systematic Reviews (PROSPERO) and following Preferred Reporting Items for Systematic Reviews and Meta-Analyses (PRISMA) principles, conducted an extensive electronic search in databases such as PubMed, MEDLINE, Cochrane, Web of Science, and Scopus. Using Medical Subject Headings (MeSH) terms and different types of studies, the search method focused on studies that directly looked at how nutrition and compression therapy affected the healing of venous ulcers. After deduplicating and screening publications, a collaborative full-text review was conducted to determine their inclusion. As a result, several research studies were chosen for the qualitative synthesis. The authors created a data extraction form to document important variables such as demographics, therapy specifics, and wound features. Several studies on patients with venous ulcers have shown that consuming basic nutrients can improve wound healing. Treatment results differed depending on the types of compression and pressure intensity. Although minimal data indicates the possible benefits of two-layer therapy, a definitive comparison is still uncertain. Further clinical studies are necessary to investigate a wider range of dietary factors and to evaluate different treatments in similar situations.

## Introduction and background

Venous ulcers, also known as chronic sores, are open wounds that commonly develop on the lower legs and are frequently linked to chronic venous insufficiency. During the healing process of a wound, you will notice different stages of improvement. It is crucial to acknowledge that the healing process may differ across individuals, and it is advisable to seek guidance from a healthcare professional for accurate diagnosis and treatment. Factors like compression treatment and an individual's dietary health can affect the healing of these ulcers. The healing of venous ulcers can vary significantly depending on factors such as the ulcer's severity, the individual's medical problems, the quality of wound care, and the person's overall health. Venous ulcers typically result from insufficient blood flow in the veins, leading to poor circulation and tissue injury, primarily in the lower legs and ankles [1].

A proper diet is crucial for aiding in the healing of wounds. Malnourishment can greatly hinder this process and raise the chances of problems. Proteins, vitamins (especially vitamins C and A), minerals (including zinc and copper), and essential fatty acids are crucial for promoting good wound healing. These nutrients have a crucial role in many phases of the healing process, including inflammation, proliferation, and remodeling. Furthermore, some studies have emphasized the need to provide nutritional support to accelerate wound healing and reduce related hazards. Ensuring sufficient intake of these nutrients is crucial for maximizing the body's capacity to heal wounds efficiently [2].

Compression therapy is the main treatment strategy for venous ulcers. This approach entails applying pressure to the injured limb using bandages or specialized garments. Compression therapy is advantageous since it improves venous blood circulation, decreases edema, and assists in wound healing [3,4].

This systematic review aimed to offer a thorough summary of the current information on how nutrition status and compression therapy affect the healing of venous ulcers. The research will compile and evaluate studies on factors affecting wound healing, including essential nutrients, the impact of malnutrition on healing, the efficacy of compression therapy methods, and the combined benefits of nutrition and compression therapy.

## Review

Materials and methods

Literature Search

**Figure 1:**
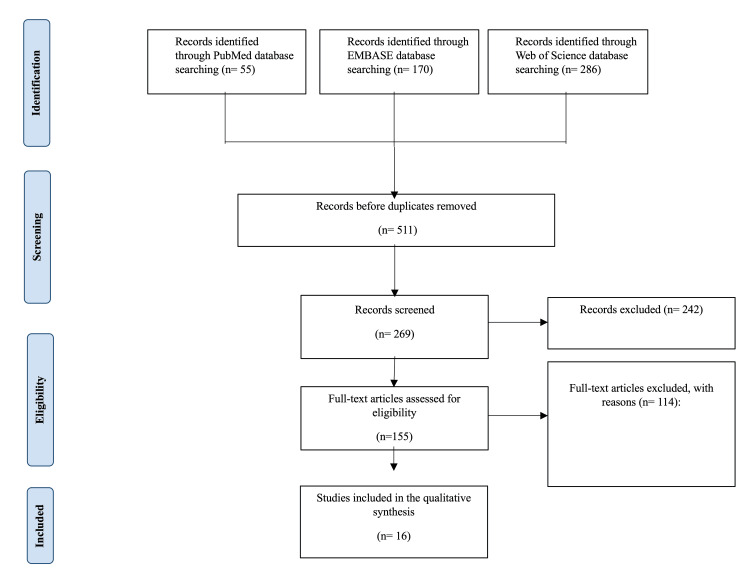
Detailed PRISMA chart used for this systematic review, outlining the many stages of the study selection process. The search technique included searching several databases as follows: PubMed (n=55), Embase (n=170), and Web of Science (n=286). At first, the records were checked for duplicates, leaving 511 distinct records. During the eligibility phase, 26 records were reviewed and 242 records were eliminated based on established criteria. Out of the records reviewed, 25 full-text articles were evaluated for eligibility, excluding 14 articles with indicated reasons. Twelve papers met the criteria for inclusion in the qualitative synthesis. PRISMA: Preferred Reporting Items for Systematic Reviews and Meta-Analyses

The systematic review was registered in PROSPERO (ID: CRD42023443967) and was conducted according to the Preferred Reporting Items for Systematic Reviews and Meta-Analysis (PRISMA) guidelines. A comprehensive electronic search was conducted using the following databases: PubMed, MEDLINE, Cochrane, Web of Science, and Scopus, with no specific time frame. The rest of the research team (AA and RA) has given their approval to a search strategy that developed. Studies related to the impact of the nutritional condition and compression treatment on the recovery of venous ulcers were identified inclusively using a combination of Medical Subject Headings (MeSH) such as "Varicose ulcer," "Venous leg ulcer," "Compression bandages," "Compression therapy," "Nutrition assessment," "Malnutrition," and "Undernutrition." In order to identify any missing articles, a further review of the references to the studies was conducted. The full search strategy with further information can be found in Figure 1.

Study Selection

The review included research investigating the influence of nutritional conditions (such as nutritional status, dietary variables, and nutrient consumption) and compression treatment on venous ulcer healing. Furthermore, research includes individuals diagnosed with venous ulcers of any degree or stage. The review analyzed different research methodologies, such as randomized controlled trials (RCTs), quasi-experimental studies, cohort studies, case-control studies, and observational studies. The review exclusively considered papers published in peer-reviewed journals or other reputable sources and written in English. Studies not focusing on the impact of dietary status and compression treatment on venous ulcer recovery were eliminated. Furthermore, research that included patients with non-venous ulcers or other wound types, animal experiments, in vitro studies, and review articles were not included. Studies that had inadequate data or were missing relevant outcome indicators were excluded from the review.

Screening and Data Extraction

After conducting the primary search, the records were imported to Google Drive (Mountain View, CA: Google) and Mendeley Desktop (London, UK: Mendeley Ltd.), where duplicate articles were removed. The remaining results were then imported into Rayyan (Doha, Qatar: Qatar Computing Research Institute) for screening by three authors (LA, SA, and RK) based on relevance determined by titles and abstracts. Next, the full texts of the studies that passed the initial screening were reviewed by two authors (EA and RA) for the final inclusion or exclusion decision. Any disagreements during the screening process were resolved through discussion with AA and the group of researchers. The data were collected from the chosen studies using an Excel spreadsheet, which included various details such as title, author, country, publication year, journal name, study design, sample size, age, gender, therapy type, initial ulcer size, active ulcer history, pain, healed wounds, wound reduction, therapy tolerance, nutritional assessment methods, body mass index, and nutrition disorders (appendix 1). We will do a data analysis and consider conducting a meta-analysis if feasible.

Statistical Analysis of Data

Despite a basic descriptive statistical analysis being performed, a meta-analysis was not possible due to the heterogeneity reported in the included publications. The Cochran's Q test indicated significant variability among the trials. The I² statistic showed a significant amount of diversity in effect sizes. Moreover, the absence of data in a suitable format for meta-analysis made it impossible to proceed.

Results

Overview of the Literature

In the initial search, we found a significant number of publications, including 55 from PubMed, 170 from Embase, and 286 from the Web of Science database. Before eliminating duplicates, we had 511 articles. After eliminating duplicates, 268 papers underwent scrutiny based on their title and abstracts, resulting in the elimination of 242 articles. Further refining was done by evaluating 26 articles in their entirety. Only 12 articles that fulfilled all the required criteria were included in this systematic review [5-15]. Fourteen papers were excluded during the full-text examination for various reasons. The exclusions were made due to issues like incomplete text availability, duplicate content, methodological flaws, irrelevant outcomes, the inclusion of patients without the studied disease, and language limitations with non-English papers. Table 1 presents an extensive compilation of research articles focusing on the impact of nutrition supplements and compression treatments on the healing of venous ulcers.

**Table 1:** Features and results of studies investigating nutritional conditions and compression therapy in venous ulcer recovery.

Studies	Country	Study design	Total patients received with venous ulcer	Total patients in the study	Modality of compression and nutritional supplements	Clinical recommendations	Level of evidence
De Franciscis et al. [5]	Italy	Observational interventional study	87	58	Modality of compression - stockings. Nutritional supplements: patients with hyperhomocysteinemia were administered folic acid therapy as part of their treatment protocol.	Hyperhomocysteinemia (HHcy) is seen as a possible indicator of chronic venous ulcer (CVU). Administering folic acid may improve wound healing in people with HHcy and venous leg ulcers.	III
Pieper and Templin [6]	United States	Cross-sectional pilot study	273	189	Modality of compression - stockings. Nutritional supplements - the study utilized two main tools for nutrition evaluation: the United States Department of Agriculture Adult Food Sufficiency Questionnaire (FSQ) and the Nestle Mini Nutritional Assessment (MNA). The measures were utilized to assess food security, eating patterns, and the likelihood of malnutrition or malnourishment among participants.	Evaluating nutrition is crucial in the care of individuals with injection-related venous ulcers. Nutritional deficits can hinder wound healing by impacting fibroblast proliferation, collagen synthesis, and epithelialization, which can result in higher care expenses.	IV
Wipke-Tevis and Stotts [7]	Columbia	Prospective study	31	26	Modality of compression - stockings and bandages. Nutritional supplements - participants did not receive any specific nutritional supplements as part of the study intervention.	Several venous ulcer patients exhibited insufficient consumption of calories, protein, and zinc, all crucial for the healing of wounds. Also, they exhibited a significant prevalence of obesity, along with some degree of depletion in the sample. Obesity and insufficient nutrition can both affect wound healing.	II
Nunes et al. [8]	Brazil	Randomized controlled trial	118	82	Modality of compression - Unna boot (Viscopaste; Smith-Nephew) and cellulose membrane (Membracel; Vuelo Pharma). Nutritional supplements - utilization of oral nutrition supplements with Cubitan, which is manufactured by Danone Nutricia.	Utilizing educational programs, physical activity, leg elevation, oral nutritional supplementation, and suitable topical and compression therapy can effectively cure venous leg ulcers and alleviate pain.	III
Wissing et al. [9]	Sweden	Case report	6	6	Modality of compression - bandages and stockings. Nutritional supplements - the trial included a personalized nutritional support regimen that incorporated liquid dietary supplements. The supplements include vital elements such vitamins, minerals, proteins, and calories to aid in wound healing and maintain overall nutritional health.	Utilizing a personalized nutritional support program, which incorporates liquid food supplements, could be advantageous for individuals with therapy-resistant venous leg ulcers. Perform a thorough nutritional evaluation for older individuals with leg ulcers, particularly if malnutrition is suspected.	V
Mościcka et al. [10]	Poland	Prospective study	35	32	Modality of compression - short-stretch bandages. Nutritional supplements - the patients underwent a customized therapy regimen that involved receiving supplementation with an energy-dense, high-protein oral formula named Cubitan. Each patient was instructed to consume 200 mL of the formula three times a day, containing 128 kcal, 10 g of protein, and 1.5 g of L-arginine per 100 mL, as well as various macro- and micro-nutrients and vitamins.	Venous leg ulcers (VLU) heal faster when you take nutritional supplements by mouth, especially a protein- and energy-dense mix with zinc, vitamins, and arginine. An all-encompassing treatment strategy, incorporating specialized dressings, wound care, and nutritional support, plays a crucial role in promoting substantial improvement in venous leg ulcer (VLU) healing.	IV
Melo et al. [11]	Brazil	Clinical trial	81	29	Modality of compression - apply 2% polyhexamethylene biguanide solution for 15 minutes, cover with 2% papain gel or hydrogel, rayon gauze, or cotton gauze for exudate absorption, bandage the metatarsal region up to four centimeters before the patellar region with a crepe bandage, and apply short-stretch and single-layer compression bandages in the same area. Nutritional supplements - the patients were given a high-calorie and high-protein oral nutritional supplement containing specific nutrients, such as arginine, zinc, and vitamins A, C, and E, which are beneficial for healing. The study made reference to a specific product from Danone Nutricia called Cubitan. Patients with ulcers smaller than 20 cm^2^ were prescribed two bottles of the supplement daily, whereas those with ulcers of at least 20 cm^2^ were instructed to take three bottles a day.	Utilizing liquid oral nutritional supplements in hospitals can result in cost savings and provide functional advantages, including enhanced mobility and a better capacity to do everyday tasks. Nutritional supplementation led to enhanced healing conditions, resulting in a notable reduction in the injury area and the Pressure Ulcer Scale for Healing (PUSH) score.	II
Wissing and Unosson [12]	Sweden	Cross-sectional study	70	64	Modality of compression - multilayer and short-stretch compression bandaging. Nutritional supplements - the patients were given protein-rich supplements containing vitamins A, C, and E, as well as minerals such as zinc and selenium, along with arginine-enriched supplements.	Patients with leg and foot ulcers should receive thorough care, which involves evaluating their nutritional status through techniques such as the Mini Nutritional Assessment (MNA). Proactive measures should be implemented to improve the nutritional condition of patients with leg and foot ulcers before malnutrition becomes apparent. Effective pain management can enhance patients' drive for physical exercise, which is crucial for sustaining physical fitness. Dietary supplements comprising energy, proteins, minerals, and vitamins can be used to maintain and enhance functional conditions and promote ulcer healing in individuals with chronic ulcers when particular food consumption goals are not met.	III
Tobón et al. [13]	United States	Cross-sectional observational study	19	8	Modality of compression - multilayer compression bandaging. Nutritional supplements - the patients were given high-calorie supplements containing vitamins A, C, and E, as well as minerals like zinc and selenium, and enhanced with arginine.	It is recommended that overweight and obese patients with chronic venous leg ulcers (VLUs) have a nutritional assessment, which includes measuring body dimensions, analyzing foods, and checking levels of important nutrients like albumin, vitamins A and C, and zinc in the blood.	IV
Alvarez et al. [14]	United States	Randomized controlled trial	52	45	Modality of compression - intermittent pneumatic compression (IPC) combined with regular compression therapy (compression bandage). Nutritional supplements - participants did not receive any specific nutritional supplements as part of the study intervention.	Adding intermittent pneumatic compression (IPC) to regular compression therapy accelerated wound healing and decreased wound discomfort in individuals with chronic venous insufficiency and difficult-to-treat lower leg ulcers.	II
Wissing et al. [15]	Sweden	Observational, cross-sectional study	26	9	Modality of compression - bandage. Nutritional supplements - the study aimed to describe the dietary intake, eating patterns, physical activity levels, and need for help among females with venous leg ulcers.	Patients with venous leg ulcers should participate in suitable physical activities within their capacity to improve circulation and accelerate healing.	IV

The studies considered in our systematic review displayed a range of study designs. Four out of the 11 studies utilized a cross-sectional study design. One study was a randomized controlled trial (RCT), one study was a case report, another was a clinical trial, and a third was an observational interventional study, with the last study conducted prospectively. This variety in design facilitated a thorough investigation of the effects of dietary circumstances and compression treatment on venous ulcer healing. The 12 papers included in this systematic review came from various regions throughout the world. Research was carried out in Italy and Columbia, and three studies were undertaken in the United States. The remaining studies were conducted in Brazil, Sweden, and Poland, in that order. The studies included in this review were published over a range of years; the earliest was published in 1997, and the most recent study came out in 2022. Table 1 offers a detailed summary of the participants' demographics in the studies covered.

Patient Profile and Characteristics

In this systematic review, a total of 631 patients were included. A total of 422 patients were examined to investigate the impact of nutrition and compression therapy on venous ulcer healing. The remaining people were used as the control group for comparison.

A higher number of female volunteers, totaling 286, was noticed in the study compared to male participants, who totaled 136. When studying people with chronic venous ulceration (CVU) and hyperhomocysteinemia (HHcy), it is important to recognize that high homocysteine levels can result from either folate insufficiency or B12 deficiency. Among patients with persistent venous ulcers, a significant proportion (62.06%) had high homocysteine levels. Individuals with both hyperhomocysteinemia (HHcy) and standard treatment, such as folic acid supplementation, showed a notably increased recovery rate of 78.75% [5]. A lot of different research papers had different ways of judging the nutrition status of the patients who were being watched to see how nutrition and compression therapy affected the healing of venous ulcers. Significantly, 84% of the subjects were classified as being at moderate or high nutritional risk [11]. Another study discovered insufficient consumption of protein, vitamin C, and zinc in a group of Swedish female CVU patients, emphasizing the need to enhance the intake of these nutrients to aid in wound healing for patients with venous leg ulcers [15].

Patient-Reported Outcomes, and Complications

This section discusses the results related to patient-reported outcomes and the incidence of problems linked to compression therapy. No patients in the studies reported experiencing itching, dry skin, or sensations of feeling cold or warm due to the compression therapy. A study showed that seven participants had pain while being treated for venous ulcers. Effective pain control is essential for the well-being and treatment success of individuals with venous ulcers. The pain from venous ulcers can result from reasons such as venous insufficiency, tissue inflammation, changing wound dressings, and the healing process. The discomforts might impact both the patient's quality of life and their adherence to treatment and progress. Emphasizing proper pain management can reduce discomfort and increase patients' motivation to participate in physical exercise, which is crucial for maintaining good physical health and improving circulation, ultimately aiding in wound healing [14].

Quality Assessment and Bias Evaluation

The studies were assessed for quality and bias according to the rating levels of evidence and grading criteria set by the American Society of Plastic Surgeons. Three studies were categorized as level 2 evidence, suggesting they had either a prospective cohort or retrospective cohort design [7,11,14] (appendix 2). Additionally, three studies were classified as level 3 evidence, indicating a case-control design (appendix 3) [5,8,12]. Four studies were classified as level 4 evidence, indicating a cohort design [6,10,13,15], and one study was classified as level 5 evidence, indicating a case report (appendix 4) [9]. These evaluations provide insights into the overall quality and potential sources of bias in the included studies, enhancing the robustness and reliability of the reported results.

Discussion

In this systematic review, we examined several methods for evaluating the effects of nutritional status and compression therapies on the healing of venous ulcers. Our research showed a strong correlation between general nutritional conditions and improved wound healing. The impact of these parameters differed depending on the type of compression therapy, pressure intensity, and the existence of hyperhomocysteinemia (HHcy) in certain instances. Correcting deficits in vitamin B12 and folic acid significantly enhanced healing outcomes in individuals with HHcy.

Venous ulcers, often called chronic sores, can appear on the lower limbs as a result of reduced blood circulation. Factors like compression treatment and an individual's dietary health can greatly impact the healing process of these ulcers. Proper nutrition plays a pivotal role in facilitating the body's healing mechanisms. Malnutrition can hinder important processes like the development of new blood vessels, collagen production, and immunological function, all of which are essential for proper wound healing. Compression therapy works by reducing venous hypertension, which is a common contributor to the formation of venous ulcers. The primary goal of a systematic review is to methodically collect and analyze a diverse range of studies to provide a thorough summary of the current evidence on a particular topic.

The diverse range of therapies for this ailment complicates the identification of the most effective healing method. Our review indicates that nutrition has a significant role in the recovery of venous ulcers. Another study highlighted the significance of proper nutrition in the healing of venous ulcers but did not specifically evaluate the impacts of nutritional treatments. It emphasized the relationship between food security, sufficiency, nutritional status, and wound healing results. The study discovered that insufficient food security is linked to reduced motivation for physical activity, while higher nutritional assessment scores are associated with improved quality of life, increased confidence in balancing abilities, and fewer falls. These findings highlight the intricate connections between diet, nutritional status, and overall healing results [3,11]. The trial, which incorporated specialized oral nutritional supplements into a complex therapeutic regimen, demonstrated promising outcomes. Patients who were administered the distinctive combination of energy-dense protein, arginine, zinc, and vitamins experienced a notable decrease in the size of their ulcers, reducing from 26.5 cm^2^ to 14.80 cm^2^. The positive correlation between nutritional supplements and wound healing, together with enhancements in pain levels and quality of life, demonstrates the potential of these therapies to aid in the recovery of venous leg ulcers (VLU) [10].

The review's findings highlight the varying impact of compression depending on its form and pressure strength. The study comparing two treatment packages for venous leg ulcer healing provided insights into the effectiveness of various wound care methods. Both bundles incorporated exercise, elevation, and compression treatment, which are essential components of comprehensive venous ulcer care. The bundles demonstrated similar healing rates, emphasizing the importance of comprehensive regimens incorporating nutritional assistance, compression, and other wound care strategies for optimal outcomes [8]. On the other hand, various studies have shown that intermittent pneumatic compression (IPC) therapy enhanced compression therapy for venous leg ulcers (VLUs). This study showed that the addition of IPC therapy to conventional compression resulted in faster wound healing rates compared to using compression therapy alone, although it was not directly associated with nutritional treatments. Patients experienced reduced wound discomfort in the initial weeks of using IPC [16].

All of those studies demonstrate the essential role of nutritional supplements in facilitating the healing of venous ulcers. Nutritional treatments, such as specific supplements or improved food intake, can positively influence wound healing, nutritional status, and overall well-being. The results suggest that a comprehensive approach involving both wound care and proper diet positively influences the healing of venous ulcers, even though the specific mechanisms may vary.

Limitations

Our systematic review has noteworthy limitations. Most of the research analyzed was carried out in Western cultures, which may limit its applicability to other cultural or ethnic groups with distinct dietary and healthcare practices. Moreover, there was diversity in nutritional evaluation techniques, varying from self-reported consumption to biomarkers. This diversity may have affected the synthesis of results. Sometimes, the information provided on the duration, kind, and pressure gradients of compression therapy was not enough to allow for an effective comparison between trials. Some research incorporated intermittent pneumatic compression in addition to traditional approaches, whereas others did not identify additional techniques, creating a challenge in comparing protocols.

In the future, research could improve by conducting multi-center trials involving varied populations. These trials should use standardized tools to assess nutritional status and well-specified parameters for compression treatment. Supporting uniformity in these areas will help with better synthesis and the discovery of the best ways to help venous ulcers heal. Additional research is required to identify the most effective types and amounts of nutrients for supplemental therapies.

## Conclusions

This systematic review analyzed several methodologies used to study the effects of nutrition and compression treatments on the healing of venous leg ulcers. Our research emphasizes the crucial importance of certain nutrients in treating leg ulcers. It specifically points out deficits in key elements, including protein, vitamin C, zinc, folate, and vitamin B12, which have been linked to a higher occurrence of these ulcers. Getting these dietary deficiencies fixed and using different pressure therapies, like bandages, intermittent pneumatic compression (IPC), and Unna boots, may help reduce the size of venous leg ulcers. Our data show that outcomes vary depending on the type of compression treatment used and the level of pressure applied.

Our examination of nutritional factors is restricted, and although some indicators support the effectiveness of two-layer therapy, we do not have enough complete data to definitively determine the superiority of one treatment over another. Therefore, it is crucial to carry out more clinical trials that cover a wider range of dietary parameters and to compare different therapies in similar situations. Through thorough study, we can enhance our knowledge to improve treatment methods for venous leg ulcers and support the development of more efficient and tailored healthcare practices.

## Appendices

Appendix 1

The data were extracted from the selected studies through an Excel sheet, including the title, author’s name, country, year of publication, name of the journal, study design, sample size, age, gender, therapy type, initial ulcer size, active ulcer history, pain, healed wounds, wound reduction, therapy tolerance, nutritional assessment methods, body mass index, and nutrition disorders.

**Table 2:** Summary of data extracted from selected studies. PROSPERO: International Prospective Register of Systematic Reviews; HHcy: hyperhomocysteinemia; CVU: chronic venous ulcer; DETERMINE: disease, enteral intake, muscle mass, tooth loss, economic stress, reduced social contact, multiple medications, involuntary weight loss or gain, needs assistance in self-care, and elderly (above 80 years old); PUSH: Pressure Ulcer Scale for Healing

Study characteristics											Participant characteristics							Intervention details																	
Author	Country	Year of publication	Study design (randomized controlled trial, cohort study, case-control study, etc.)	Sample size			Age			Gender				Therapy type		Initial ulcer size				Active ulcer history		Pain		Healed wounds		Wound reduction		Therapy tolerance		Nutrition assessment method		Body mass index (obesity)		Nutrition disorders	
				Group A	Group B	Total	Mean	Standard deviation (median)	Range	Male		Female		Group A	Group B	Group A	Mean	Group B	Mean	Group A	Group B	Group A	Group B	Group A	Group B	Group A	Group B	Group A	Group B	Group A	Group B	Group A	Group B	Group A	Group B
										Group A	Group B	Group A	Group B			Range		Range																	
De Franciscis [5]	Italy	2015	Observational interventional study	54	33	87	N/A	67	46-83 years	17 (31.48%)	9 (27.27%)	37 (68.51%)	24 (72.73%)	CVU patients with HHcy treated with folic acid (1·2 mg/day) + basic treatment for 12 months	CVU patients without HHcy are treated only with basic treatment. Basic treatment consists of treatment with third-class medical compression stocking (36-50 mmHg) for 12 months and then with second-class medical compression stocking (25-40 mmHg) for the remaining 24 months	3.2-18.5 cm^2^	13.7 cm^2^	3.6-20.7 cm^2^	15.9 cm^2^	N/A	N/A	N/A	N/A	37 (78.72%)	19 (63.33%)	N/A	N/A	47 out of 54 patients (87.04%) completed the study and received folic acid treatment	30 out of 33 patients (90.91%) completed the study without folic acid treatment	N/A	N/A	11 (20.37%)	8 (24.24%)	N/A	N/A
Pieper and Templin [6]	United States	2015	Cross-sectional pilot study	84	189	273	61	N/A	N/A	N/A	N/A	N/A	N/A	N/A	Compression therapy	N/A	N/A	N/A	N/A	N/A	History of wound debridement, and deep ulcers (>2 cm)	N/A	N/A	N/A	N/A	N/A	N/A	N/A	N/A	United States Department of Agriculture Adult Food Sufficiency Questionnaire (FSQ)	Nestle Mini Nutritional Assessment (MNA)	N/A	High BMI (>33 kg/m^2^)	Protein deficiency	N/A
Wipke-Tevis and Stotts [7]	Columbia	1998	Prospective study	N/A	N/A	31	59.8	15.3	29-86 years	60%	N/A	40%	N/A	N/A	N/A	7.66 cm	SD=2.21	5.62 cm	SD=4.85	N/A	N/A	N/A	N/A	13%	18%	26.0% (SD =79)	27% (SD =80)	N/A	N/A	DETERMINE Public Awareness Checklist (PAC)	DETERMINE Public Awareness Checklist (PAC)	31.3 kg/m² (SD =7.3)	31.3 kg/m² (SD= 8.0)	84% of the participants had moderate or high nutritional risk	50% of the participants were classified as obese based on BMI measurements
Nunes et al. [8]	Brazil	2019	Randomized controlled trial	14	14	28	59.6	11.95	N/A	N/A	N/A	71.40%	64.30%	2% papain/ ﬁrst phase (compression therapy with Unna boot/second phase)	2% hydrogel/ﬁrst phase (cellulose membrane with Unna boot/second phase)	14.01 cm	11.5	138.71 cm	47.15	N/A	N/A	1.57 (2.70)	2.14 (3.00)	71.42%	64.28%	22.12 (SD= 67.12)	42.95 (SD= 33.83)	N/A	N/A	Providing the participants with an oral nutritional supplement called "Cubitan," manufactured by Danone Nutricia, and booklet with healthy-eating guidelines based on the recommendations of the Brazilian Ministry of Health	Providing the participants with an oral nutritional supplement called "Cubitan," manufactured by Danone Nutricia, and booklet with healthy-eating guidelines based on the recommendations of the Brazilian Ministry of Health	31.91	31.21	N/A	N/A
Wissing et al. [9]	Sweden	2022	Single-case study	6	N/A	6	84.5 ±5.7 years	N/A	79 and 93 years	2	N/A	4	N/A	Follow an individualized diet plan which includes the use of liquid dietary supplements (Fortimel, Nutricia AB)	N/A	N/A	N/A	N/A	N/A	N/A	N/A	N/A	N/A	N/A	N/A	N/A	N/A	N/A	N/A	N/A	N/A	N/A	N/A	N/A	N/A
Mościcka et al. [10]	Poland	2022	Prospective study	N/A	N/A	35	71.7	11.3	47.0-96.0	13 (37%)	N/A	N/A	N/A	Complex treatment, which included specialized wound care with dressings, along with a specialized oral nutritional supplementation	N/A	N/A	N/A	N/A	N/A	N/A	N/A	N/A	N/A	N/A	N/A	30 (86%)	N/A	30 out of 34 (88%)	N/A	Dietary history, anthropometric measurements, and, laboratory tests	N/A	13 (37.1%)	N/A	Malnutrition that impairs the wound-healing process	N/A
Melo et al. [11]	Brazil	2022	Clinical trial	N/A	N/A	27	61	11,2	N/A	N/A	N/A	N/A	N/A	Nutritional supplementation therapy	N/A	N/A	N/A	N/A	N/A	N/A	N/A	N/A	N/A	N/A	N/A	N/A	N/A	27 out of 29	N/A	Body composition and 24-hour food diaries, planigraphy of injuries and PUSH, blood sampling for biochemical tests	N/A	31.3±5.1	N/A	Protein-energy malnutrition	N/A
Wissing and Unosson [12]	Sweden	1999	Cross-sectional	N/A	N/A	70	79	6.5	N/A	20	N/A	50	N/A	N/A	N/A	N/A	N/A	N/A	N/A	Most common diagnosis was venous ulcers; more than one ulcer: 32 patients; ulcers in both legs: 14 patients; ulcer in one or both feet: 5 patients; isolated foot ulcers: 15 patients	N/A	Pain was reported by 19 out of the 36 well-nourished patients, pain was reported by 25 out of the 34 at risk of malnutrition/malnutrition patients	N/A	N/A	N/A	N/A	N/A	N/A	N/A	Mini nutritional assessment (MNA)	N/A	Men well nourished: 26.3 kg/m^2^, at risk of malnutrition:23.1 kg/m^2^, women well nourished: 27.3 kg/m^2^, at risk of malnutrition:21 kg/m^2^	N/A	32 patients at risk of malnutrition and 2 female patients are malnourished	N/A
Tobón et al. [13]	United States	2008	Cross-sectional observational design	N/A	N/A	8	65.6	10.4	53-79	6	N/A	2	N/A	N/A	N/A	1.75 cm^2^-360 cm^2^	60 cm^2^	N/A	N/A	N/A	N/A	N/A	N/A	N/A	N/A	-53.1% (median)	N/A	N/A	N/A	By age, gender in addition to observing the average food log over 3 days and testing blood for levels of albumin, vitamin A, vitamin C, and zinc	N/A	4 (50%)	N/A	N/A	N/A
Alvarez et al. [14]	United States	2020	Randomized controlled trial	27	25	52	N/A	N/A	N/A	N/A	N/A	N/A	N/A	The ulcers were dressed and bandaged using the same 4-layer bandage system described for the control group	The ulcers were dressed and bandaged using the same 4-layer bandage system described for the control group in addition to compression therapy provided by a 4-chamber intermittent gradient, sequential, pneumatic compression device	N/A	Ankle/calf, cm: 44.7/56.4	Ankle/calf, cm: 46.5/59.3	N/A	N/A	N/A	N/A	N/A	N/A	N/A	Ankle/calf, %: 12.0/13.2	Ankle/calf, %: 19.1/18.7	N/A	N/A	N/A	N/A	N/A	N/A	N/A	N/A
Wissing et al. [15]	Sweden	1997	Observational, cross-sectional study	N/A	N/A	9	N/A	N/A	55-60 years	N/A	N/A	N/A	N/A	N/A	N/A	N/A	N/A	N/A	N/A	N/A	N/A	N/A	N/A	N/A	N/A	N/A	N/A	N/A	N/A	N/A	N/A	N/A	N/A	N/A	N/A

Appendix 2

Assessment of Study Quality and Risk of Bias (Cohort Study)

For a study to be categorized as good quality it must achieve 3 or 4 stars in the selection domain, along with 1 or 2 stars in the compatibility domain, and 2 or 3 stars in the outcome/exposure domain; to be categorized as fair quality it must achieve 2 stars in the selection domain, along with 1 or 2 stars in the comparability domain, and 2 or 3 stars in the outcome/exposure domain; and to be categorized as poor quality it must receive 0 or 1 star in the selection domain, 0 stars in the comparability domain, or 0 or 1 star in the outcome/exposure domain.

**Table 3:** Evaluation of the quality of cohort studies and the risk of bias based on selection, comparability, and outcome. *A moderate level of importance. **A higher level of importance.

Studies	Selection				Comparability	Outcome			Quality score	Risk of bias (0-3: high, 4-6: moderate, and 7-9: low)
	Q1	Q2	Q3	Q4	Q5	Q6	Q7	Q8		
Wipke-Tevis and Stotts [7]	*	*	*	*	**	*	*	*	Good quality study (9)	Low risk
Mościcka et al. [10]	*	-	*	*	*	*	*	*	Good quality study (7)	Low risk
Selection: Q1: Was the exposure cohort representative of the population under study? Q2: Was the non-exposure cohort selection appropriate and representative? Q3: Was the ascertainment of exposure adequately conducted? Q4: Was there a demonstration that the outcome of interest was not present at the beginning of the study? Q5: How comparable were the cohorts based on the study design or analysis? Q6: Was the assessment of the outcome conducted appropriately? Q7: Was the follow-up duration sufficient for outcomes to manifest? Q8: Was the follow-up of cohorts deemed adequate?										

Appendix 3

Assessment of Risk of Bias (Randomized Controlled Trial)

For a study to be categorized as good quality, it must achieve 4 or 5 stars in selection domain, 1 or 2 stars in compatibility domain, and 2 or 3 stars in outcome/exposure domain; to be categorized as fair quality, it must achieve 2 or 3 stars in selection domain, 1 or 2 stars in comparability domain, and 2 or 3 stars in outcome/exposure domain; and to be categorized as poor quality, it must achieve 0 or 1 star in selection domain, 0 stars in comparability domain, or 0 or 1 stars in outcome/exposure domain.

**Table 4:** Evaluation of bias in randomized controlled trials is based on the randomization process, deviations from intended interventions, participant and personnel blinding, missing outcome data, outcome measurement, selection of reported results, and the overall risk of bias.

Studies	Bias arising from the randomization process	Bias due to deviations from intended interventions	Blinding of participants and personal	Bias due to missing outcome data	Bias in measurement of the outcome	Bias in selection of the reported result	Overall RoB
De Franciscis [5]	Unclear	Unclear	Low	Low	Low	Low	Low
Nunes et al. [8]	Low	Low	Low	Low	Low	Low	Low
Melo et al. [11]	Unclear	Unclear	Unclear	Unclear	Low	Low	High

Appendix 4

Assessment of Study Quality and Risk of Bias (Cross-Sectional Study)

The following are accepted thresholds for converting the Newcastle-Ottawa scales to Agency for Healthcare Research and Quality (AHRQ) standards (good, fair, and poor quality studies): for a study to be categorized as good quality, it must achieve 3 or 4 stars in selection domain, 1 or 2 stars in compatibility domain, 2 or 3 stars in outcome/exposure domain; to be categorized as fair quality, it must achieve 2 stars in selection domain, 1 or 2 stars in comparability domain, and 2 or 3 stars in outcome/exposure domain; and to be categorized as poor quality, it must achieve 0 or 1 star in selection domain, 0 stars in comparability domain, or 0 or 1 stars in outcome/exposure domain.

**Table 5:** Evaluation of the quality of cross-sectional studies and the risk of bias based on selection, comparability, and outcome. *A moderate level of importance. **A higher level of importance.

Author	Selection					Comparability	Outcome		Quality score	Risk of bias (0-3: high, 4-6: moderate, and 7-9: low)
	Q1	Q2	Q3	Q4	Q5	Q6	Q7	Q8		
Pieper and Templin [6]	*	-		* *		*	**	*	Fair quality study (6)	Low risk
Tobón et al. [13]	-	*	*	**		*	**	*	Fair quality study (7)	Low risk
Selection: Q1: Was the exposure cohort representative of the target population? Q2: Was the sample size appropriate for the study objectives? Q3: Was the ascertainment of exposure adequately performed? Q4: What was the response rate of the study participants Q5: How was the screening/surveillance tool ascertained? Comparability: Q6: Were potential confounders investigated through subgroup analysis or multivariable analysis? Outcome: Q7: How was the outcome assessed? Q8: Which statistical tests were used for analyzing the data?										
