# Correction: The Impact of Nutritional Condition and Compression Treatment on Venous Ulcer Recovery: A Systematic Review

**DOI:** 10.7759/cureus.c263

**Published:** 2025-08-26

**Authors:** Arwa T Alsharif, Omar I Alanazi, Rayan A Alqarni, Hawazen O Alahmadi, Lamis A Alassiri, Shahad A Alamri, Razan M Khalifa, Eman A Alshehab, Rahmah B Alzahrani, Abdullah Al Wahbi

**Affiliations:** 1 Department of Medicine, Batterjee Medical College, Jeddah, SAU; 2 Department of Medicine, King Saud Bin Abdulaziz University for Health Sciences, Riyadh, SAU; 3 Department of Medicine, Imam Mohammad Ibn Saud Islamic University (IMSIU), Riyadh, SAU; 4 Department of Medicine, Taibah University, Medina, SAU; 5 Department of Medicine, King Abdulaziz University, Jeddah, SAU; 6 Department of Medicine, Umm Al-Qura University, Mecca, SAU; 7 Department of Clinical Nutrition, King Fahd Hospital, Medina, SAU; 8 Department of Vascular Surgery, King Abdulaziz Medical City Riyadh, Riyadh, SAU; 9 Department of Surgery, King Saud University for Health Sciences, Riyadh, SAU

This article has been corrected to fix the following typos in the PRISMA diagram:

- "Records screened (n=268)" corrected to "Records screened (n=269)"
- "Full-text articles assessed for eligibility (n=26)" corrected to "Full-text articles assessed for eligibility (n=155)"
- "Full-text articles excluded, with reasons (n=14)" corrected to "Full-text articles excluded, with reasons (n=114)"
- "Studies included in the qualitative synthesis (n=12)" corrected to "Studies included in the qualitative synthesis (n=16)"

